# Left Atrial Appendage Thrombosis and Oral Anticoagulants: A Meta-Analysis of Risk and Treatment Response

**DOI:** 10.3390/jcdd9100351

**Published:** 2022-10-13

**Authors:** Yun-Yung Cheng, Shennie Tan, Chien-Tai Hong, Cheng-Chang Yang, Lung Chan

**Affiliations:** 1 Department of Neurology, Shuang-Ho Hospital, Taipei Medical University, New Taipei 235, Taiwan; 2 Department of Neurology, School of Medicine, College of Medicine, Taipei Medical University, Taipei 235, Taiwan; 3 Brain and Consciousness Research Center, Shuang Ho Hospital, Taipei Medical University, New Taipei 235, Taiwan

**Keywords:** left atrial appendage thrombus, atrial fibrillation, stroke, oral anticoagulant

## Abstract

Left atrial appendage thrombus (LAAT) is the main cause of cardioembolism in patients with nonvalvular atrial fibrillation (AF). Emerging evidence indicates that direct oral anticoagulants (DOACs) may be a preferred, safer choice for patients with LAAT. However, current guidelines indicate vitamin K antagonist (VKA) as the preferred treatment for LAAT. We conducted a meta-analysis to compare the efficacy of VKA and DOAC for the treatment of LAAT. **Methods:** The search was conducted in the PubMed, Embase, Google Scholar, and Cochrane Library databases from inception to July 2022, with the language restricted to English. A first analysis was conducted to evaluate the risk of LAAT under VKA or DOAC treatment. A second analysis was conducted to compare the resolution of LAAT under VKA and DOAC treatment. **Results:** In 13 studies comparing LAAT incidence rates under VKA and DOAC treatment, significant superiority of DOAC was detected (pooled RR = 0.65, 95% CI = 0.47–0.90, *p* = 0.009) with moderate heterogeneity being identified in the pooled studies. In 13 studies comparing LAAT resolution under VKA and DOAC use, treatment with DOAC exhibited a significantly increased probability of LAAT resolution compared with VKA (pooled odds ratio = 1.52, 95% CI = 1.02–2.26, *p* = 0.040). **Conclusions:** This meta-analysis suggests a superiority of DOAC over VKA with respect to LAAT incidence in people with AF and the likelihood of LAAT resolution. Due to their established safety profile, DOAC is a preferable choice for anticoagulation, although further randomized controlled studies are warranted to provide further evidence of their suitability as a new recommended treatment.

## 1. Introduction

Stroke is the leading cause of death and disability worldwide [1]. Ischemic stroke, which accounts for more than 70% of the overall incidence of stroke in developed countries, has various causes, such as large artery atherosclerosis in cerebral circulation, occlusion of cerebral small vessels, and cardiac embolism [2]. Of these causes, cardiac embolism contributes most to the increasing incidence of ischemic stroke [3]. Atrial fibrillation (AF) also independently contributes to the increased occurrence of ischemic stroke and is the most common sustained arrhythmia in older adults. In nonvalvular AF, the left atrial appendage (LAA) is the location most susceptible to thrombus formation, accounting for more than 90% of cases [4].

Even though LAA is the prime location of thrombus formation in AF patients, accumulative evidence shows that LAA thrombus may also occur in patients with sinus rhythm or even subclinical AF [5]. Through the advances and widespread use of medical devices, more cryptogenic strokes have been found to be related to subclinical AF [6].

Identifying the cause of stroke is vital in achieving optimal therapeutic strategies for the treatment and prevention of recurrent stroke [7]. Initiation of anticoagulation therapy with vitamin K antagonist (VKA) is the most common and conventional strategy employed for LAA thrombus (LAAT) [8]. However, these practices are slowly changing after the launch of the nonvitamin K direct oral anticoagulant (DOAC) in 2002. The introduction of the IIa inhibitor, dabigatran, and Xa inhibitors rivaroxaban, apixaban, and edoxaban in the millennium year has proved that these anticoagulants were at least as effective as VKA in AF for stroke prevention [9].

The safety profiles of DOAC have been highly recognized in many meta-analytic studies and healthcare databases [10,11]. Given that VKA requires regular coagulation monitoring and the potential effects from its interactions with drugs and food [12], DOAC’s high efficacy and reliable safety profile are preferred over VKA in current clinical settings. Therefore, DOAC is now generally accepted as the treatment of choice over VKA in patients with nonvalvular AF [11,13].

This trend of switching from VKA to DOAC is not limited to the prevention of strokes from nonvalvular AF; it also extends to other forms of thromboembolism, such as deep venous thrombosis. Moreover, many emerging studies assessed the comparability of DOAC against VKA for LAAT prevention and resolution. However, the optimal treatment for LAAT is yet to be established.

To the best of our knowledge, not many large-scale randomized controlled trials have attempted to verify the differences between the roles of VKA and DOAC in the risk of LAAT formation and rate of thrombus resolution. Furthermore, the lack of large-scale cohort studies has impeded guidelines from being developed that would provide high-level recommendations for LAAT medication management.

The present study is a systematic review of the outcomes of VKA and DOAC use and was performed through an examination of real-world evidence. Further, a meta-analysis of available data was also performed to compare the effectiveness of VKA and DOAC for primary prevention and resolution of LAAT. In this meta-analysis, we included studies providing specific data on the incidence of LAAT and the LAAT resolution rate under VKA or DOAC use.

## 2. Methods

### 2.1. Research Question and Objectives

In this meta-analysis, we aimed to synthesize evidence to systematically review real-world evidence for a comparison of VKAs and DOAC with respect to their influence on the (i) risk of LAAT and (ii) resolution of LAAT.

### 2.2. Selection of Articles

Relevant studies, including case series and clinical trials published before July 2022 were identified from the PubMed, Embase, Google Scholar, and Cochrane databases. Only publications in English were included. We used the following sets of terms in our search: (warfarin (Title/Abstract)) OR (novel oral anticoagulant (Title/Abstract)) OR (oral anticoagulant (Title/Abstract)) OR (anticoagulant (Title/Abstract)) OR (direct oral anticoagulant (Title/Abstract)) OR (vitamin K anticoagulant (Title/Abstract)) OR (non-vitamin K oral anticoagulant (Title/Abstract)) OR (dabigatran (Title/Abstract)) OR (rivaroxaban (Title/Abstract)) OR (apixaban (Title/Abstract)) OR (edoxaban (Title/Abstract)) AND (left atrial appendage thrombus (Title/Abstract)) OR (left atrial thrombus (Title/Abstract)).

**Figure 1:**
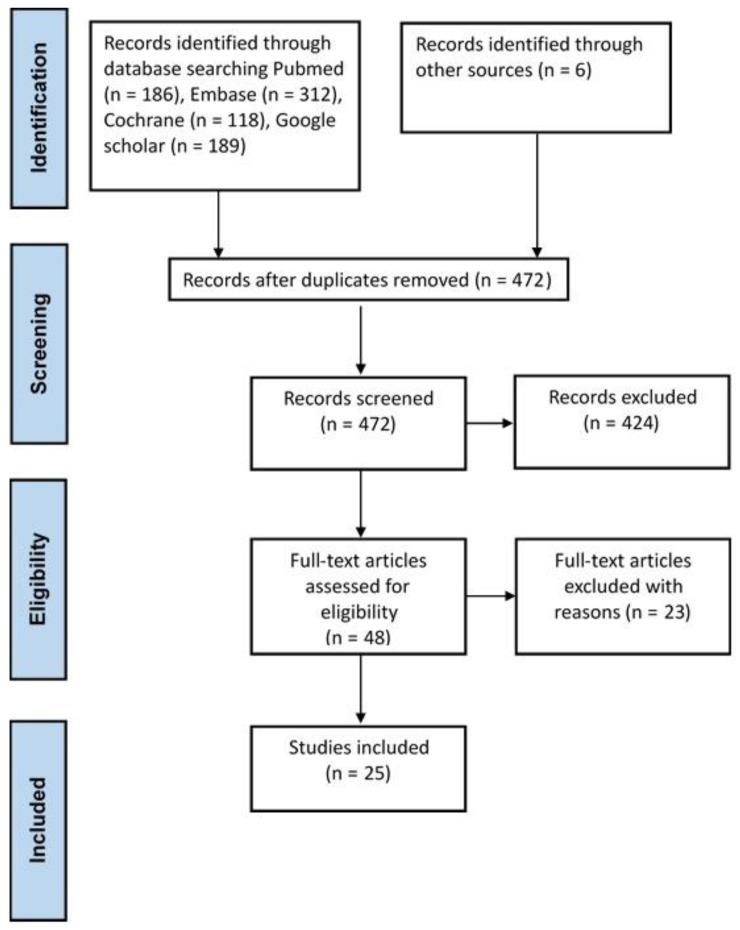
Flow diagram of study selection and search results.

The syntax used in the database searches is detailed in the Supplementary Information (Table S1). Duplicate articles from different databases were excluded. The selection process is illustrated in Figure 1. All search records from all databases were downloaded and merged into Endnote.

### 2.3. Study Design

We included studies (1) with LAA thrombus diagnosed using transesophageal echocardiography (TEE); (2) with clear records of VKA or DOAC anticoagulant use and in which patients were appropriately anticoagulated; (3) that were cohort studies published as original articles, or case series; and (4) that were publications in English.

### 2.4. Data Extraction

The selected studies were independently retrieved by two reviewers (Y-Y.C. and C-C.Y.) and were further reviewed by another author (S.T.). The selected studies were reviewed to identify the type of study, year of publication, total patient population, mean patient age, percentage of patients taking VKA, percentage of patients taking DOAC, percentage of male participants, and mean duration of anticoagulant use. Any disagreements were resolved by a fourth reviewer (C-T.H.).

## 3. Outcomes

The efficacy of the primary prevention method was evaluated based on the risk of LAAT. A second comparison was made of the potency of VKA and DOAC in resolving LAAT.

### 3.1. Synthesis of Results and Measures of Inconsistency

A random-effects model was implemented to assess LAAT incidence and resolution under VKA and DOAC use. *Q* and *I*^2^ were used to assess the level of heterogeneity between the included studies [14]. *Q* is a measure of the weighted sum of the squared deviations of the effect size of each study from the overall mean effect size and thereby serves as a test of heterogeneity significance (*p* ≤ 0.05) [15]. *I*^2^ is a measure of relative heterogeneity, estimating the percentage of the variability of effect estimates that occurs due to heterogeneity rather than due to chance. *I*^2^ ranges from 0% to 100%, with a value of 0% indicating no observed heterogeneity and a value greater than 50% representing moderate heterogeneity [16]. Furthermore, tau-squared (*T*^2^) measures the variance of the true effect as an estimate of absolute heterogeneity in effect sizes. When the observed variance increases or when the variance within studies decreases, *T*^2^ increases accordingly [15].

### 3.2. Publication Bias

We used funnel plots [17], Egger’s test [18], and the Begg and Mazumdar rank correlation test [19] to assess publication bias.

### 3.3. Statistical Analysis

All statistical analyses were performed using Stata 17. The meta-analysis is registered with PROSPERO (CRD42022319759) and was performed in accordance with the Preferred Reporting Items for Systematic Review and Meta-Analyses (PRISMA) guidelines [20]. Standard deviation was calculated using the provided confidence interval (CI) limits, standard errors, or interquartile ranges. The overall risk/odds ratios were pooled using a random-effects model. Publication bias was assessed using the funnel plot of each study’s effect size against precision (1/SE). Publication bias was investigated using Egger’s test at *p* < 0.10.

## 4. Results

### 4.1. Study Selection

After removing duplicate studies, we identified 811 articles for screening. After the exclusion of ineligible studies, 48 studies were included in the full-article assessment, and additional 23 studies were excluded because they were case reports, animal studies, or did not contain original data. Finally, 25 studies were included for qualitative synthesis. We further segregated the 25 studies into two categories: (1) cross-sectional risk analysis of developing LAAT under VKA or DOAC use and (2) analysis of LAAT resolution under VKA or DOAC use.

### 4.2. Study Characteristics

The characteristics of the included studies on the incidence of LAAT under VKA or DOAC use are listed in Table 1, and the characteristics of those on the LAAT resolution rate under VKA or DOAC use are listed in Table 2. 13 studies were cross-sectional analyses of the incidence of LAAT under VKA and DOAC use (involving 8609 individuals), and 13 were longitudinal analyses of the LAAT resolution rate under VKA and DOAC use (involving 922 individuals).

### 4.3. LAAT Incidence under VKA and DOAC

A total of 13 cross-sectional analyses involving 8609 individuals were included in the first meta-analysis of the associations between VKA and DOAC use with LAAT. These studies were published between 2016 and 2022. Among the studies, four were conducted in Poland [27,29,30,33], two were conducted in Japan [25,32], two were conducted in the United States [24,28], and one was conducted each in the EU [26], Germany [31], Canada [21], Turkey [23], and Italy [22]. The selected studies were mostly conducted in Western countries, and only two were conducted in an Asian population (Japan). Six studies were prospective [21,22,23,26,29,33], and seven were retrospective [24,25,27,28,30,31,32].

Only the ENSURE-AF trial was a randomized, multicenter, global investigation [26]. The others were mainly single-center studies. Generally, patients were considered sufficiently anticoagulated after at least 3 or 4 weeks of administration of VKA or DOAC. TEE was performed for all study participants to detect the presence of LAAT. The allocation methods for VKA and DOAC were based on the clinician’s decision.

The studies included different parameters for predicting the risk of LAAT. A higher CHA2DS2-VASc score [22,23,25,33], reduced left ventricular ejection fraction [22,23,27,33], reduced left atrial flow velocity [23,25,30,32], reduced B-type natriuretic peptide, and larger left atrium [25] were associated with the risk of LAAT. The demographic predictors of the risk of LAAT included aging, a lower body weight, lower creatinine clearance, heart failure, and diuretic treatment were also listed. [26].

### 4.4. LAAT Resolution Rate under VKA and DOAC Use

A total of 13 studies involving 922 patients were included in the second meta-analysis. These studies included three in Japan [25,38,40], five in European countries [30,37,39,43,44], three in China [34,36,42], and two in the United States [35,41]. One of the studies, the X-TRA study, was a multinational large-scale, prospective, single-arm, open-label, multicenter study. The X-TRA study evaluated a 6-week rivaroxaban treatment for left atrial and LAA thrombus resolution. Another study, the CLOT-AF study, retrospectively examined standard anticoagulation care provided to patients with left atrial and LAA thrombus for 3 to 12 weeks. These studies were published between 2015 and 2022. When the included study period was extended to 7 months, nine patients were identified as having LAAT [42]. When the included duration and dosage of anticoagulation were increased and considering the transition to DOAC, 12 patients (5%) were identified as experiencing LAAT resolution [41]. Of the 13 included studies, Kawabata, Karwowski, Durmaz, Feickert, and Shiraki studies contained data on the use of dabigatran, rivaroxaban, apixaban, and edoxaban, respectively. Edoxaban was used less frequently than the other three DOACs.

### 4.5. LAAT Incidence under VKA and DOAC Use

**Figure 2:**
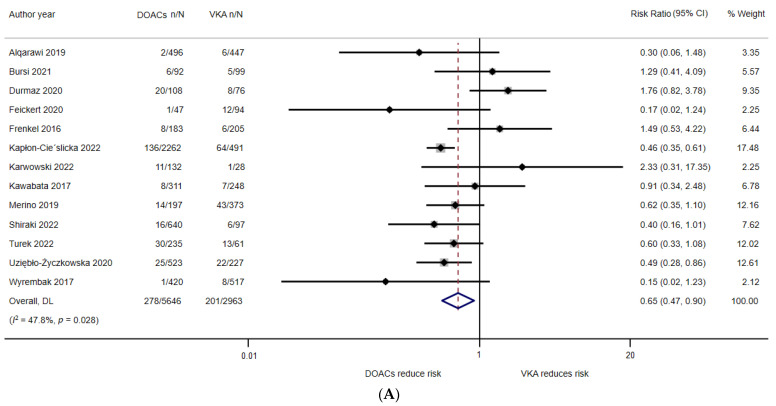

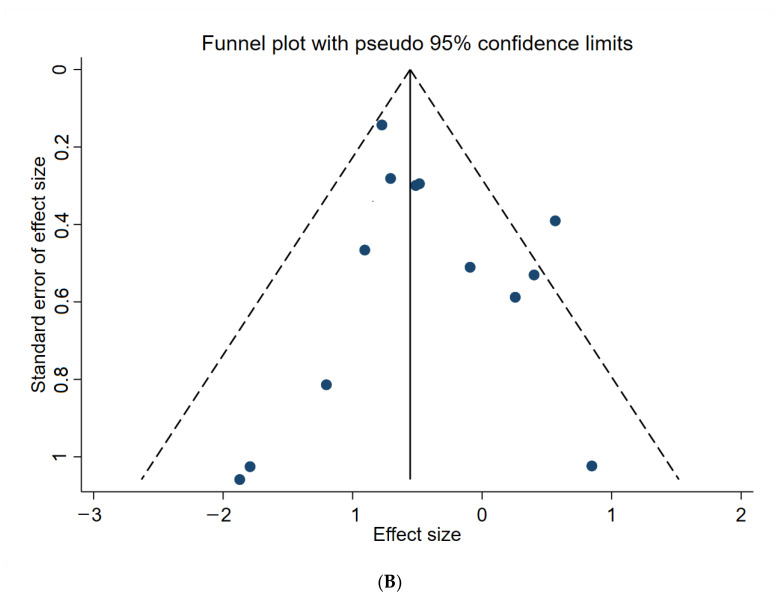
(**A**) The forest plot of random-effect meta-analysis of the incidence of left arterial appendage thrombus formation under the use of vitamin K antagonist (VKA) and direct oral anticoagulants (DOACs). (**B**) The funnel plot of the studies included in the meta-analysis of the incidence of left arterial appendage thrombus formation under the use of vitamin K antagonist (VKA) and direct oral anticoagulants (DOACs).

In the meta-analysis, 2963 patients were in the VKA arm, and 5646 were in the DOAC arm. The overall risk of LAAT under either VKA or DOAC treatment was 5.56% (479/8609). The risk ratio was derived from individual studies. The respective relative risks (RRs) with 95% CIs are listed on the right side of the forest plot (Figure 2A). The meta-analysis of 13 studies on LAAT incidence revealed significant superiority for DOACs (pooled RR = 0.65, 95% CI = 0.47–0.90, *p* = 0.009). Nearly moderate and significant heterogeneity (*Q*_12_ = 22.97, *p* = 0.028; *I*^2^ = 47.8%; *T*^2^ = 0.13) was identified. The funnel plot (Figure 2B) revealed symmetric distribution. Egger’s test (intercept = 0.684, t = 0.97, 2-tailed *p* = 0.352) and Begg’s test (z = 0.18, *p* = 0.855) did not reveal any publication bias. The meta-regression analysis showed that none of the between-study variables significantly predicted the LAAT incidence under VKA and DOAC use (mean age of participants: β = 0.071, *p* = 0.333; male ratio: β = 0.354, *p* = 0.808).

### 4.6. LAAT Resolution Rate under VKA and DOAC Use

**Figure 3:**
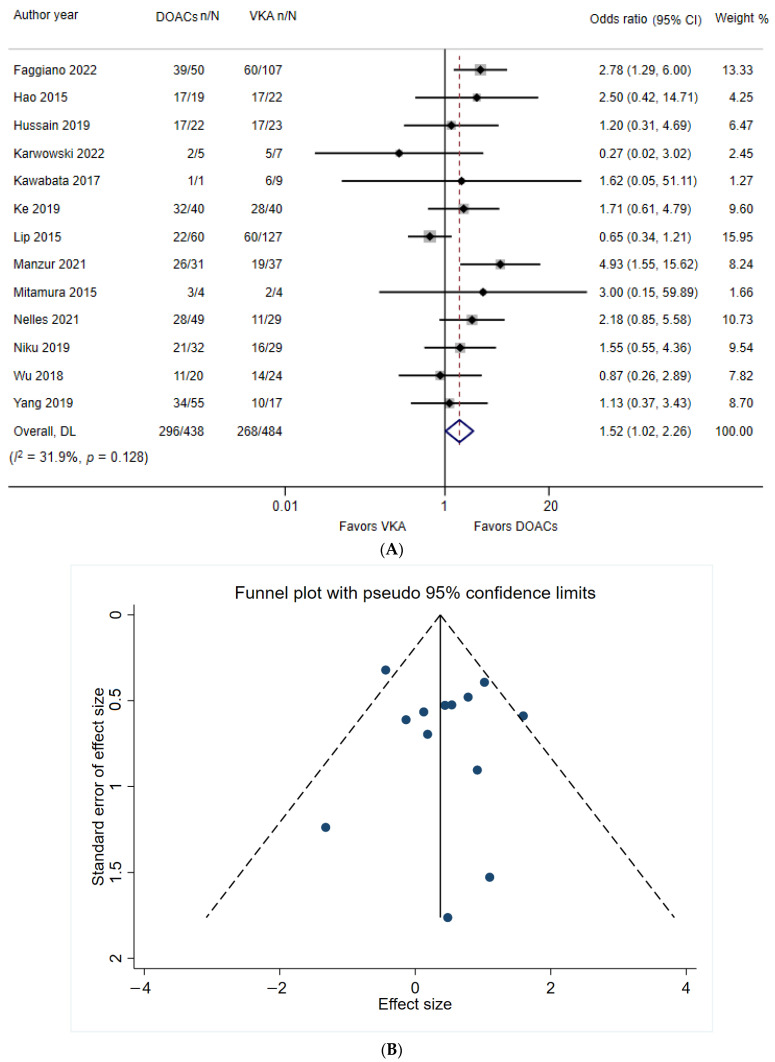
(**A**) The forest plot of random-effect meta-analysis of the likelihood of resolution of left arterial appendage thrombus under the use of VKA and DOACs. (**B**) The funnel plot of the studies included in the meta-analysis of the likelihood of resolution of left arterial appendage thrombus under the use of VKA and DOACs.

The VKA and DOAC arms included 484 and 438 patients, respectively. The summarized mean percentages of LAAT resolution for VKA and DOAC were 55.4% (268/484) and 67.6% (296/438), respectively. The odds ratio was derived from the individual studies (Figure 3A). This meta-analysis revealed that DOAC significantly increased the probability of LAAT resolution compared with VKA (pooled odds ratio = 1.52, 95% CI = 1.02–2.26, *p* = 0.040). In addition, no significant heterogeneity (*Q*_12_ = 17.62, *p* = 0.128; *I*^2^ = 31.9%; *T*^2^ = 0.16) was identified. The funnel plot (Figure 3B), although slightly asymmetric, did not indicate a high risk of publication bias. Egger’s test (intercept = 0.383, t = 0.44, 2-tailed *p* = 0.671) and Begg’s test (z = 0.55, *p* = 0.583) did not reveal any publication bias. The meta-regression analysis showed that none of the between-study variables significantly predicted the LAAT resolution rate for VKA and DOAC (mean age of participants: β = −0.042, *p* = 0.343; male ratio: β = 0.248, *p* = 0.860).

## 5. Discussion

Various guidelines recommend DOAC as a preferable anticoagulant option to VKA for stroke prevention in AF. However, evidence on optimal anticoagulant selection in patients with LAAT is lacking. Although DOACs are safer than VKAs, their efficacy remains unverified. After analyzing pooled data from studies conducted in the past decade, we discovered that DOACs showed superiority over VKAs in increasing the likelihood of LAAT resolution and reducing the chances of LAAT development in high-risk patients. By monitoring patients taking anticoagulants, the incidence of thrombus formation in patients taking DOACs was lower than in those taking VKAs. In addition, patients with LAAT showed a higher thrombus resolution rate with DOACs use in comparison to VKAs. The superiority of the safety of DOAC compared with VKA is well established; these findings provide evidence for developing future treatment recommendations.

AF is associated with a high incidence of LAAT [45]. Previous reports have demonstrated that the CHADS2 score is an independent predictor of LAAT, and the prevalence of LAAT increases with the CHADS2 score [46,47]. However, this parameter does not have a significant skew; therefore, its exclusion from this meta-analysis did not affect the risk of LAAT and resolution obtained from each included study.

Clinically, when the efficacy of anticoagulants in LAAT resolution is comparable, safety concerns, including the risk of bleeding and drug–drug interactions, become the priority of medicine choice. DOACs have not yet been approved for patients with mechanical mitral valves, thrombus in locations other than the LAA, and antiphospholipid syndrome. However, a growing body of evidence has demonstrated that DOACs lead to fewer bleeding complications than VKAs. Therefore, DOACs are more likely to be selected for the prevention of thromboembolism events in people with LAAT.

Many large-scale clinical phase III trials have demonstrated that the efficacy of DOACs in preventing stroke is superior to VKAs and that DOACs have lower rates of bleeding. However, conclusive data on the recommended type and duration of anticoagulant use in LAAT is limited. Two recent meta-analyses have demonstrated that DOACs are as efficacious as and safer than VKAs in the treatment of LAAT in patients with nonvalvular AF. In addition, two ongoing prospective randomized trial registry studies are seeking to compare DOAC and VKA in patients with LAAT. One of these is a randomized control trial in China (NCT03792152), in which the effectiveness of rivaroxaban and VKA are compared. The other randomized control trial (RE-LATED AF (NCT02256683)) is a comparison of dabigatran and VKA in patients with nonvalvular AF of LAAT.

The strength of this study is its analyses of both the risk of LAAT development in high-risk patients and the likelihood of LAAT resolution with the use of VKAs and DOACs. The development of LAAT is an indicator of subsequent systemic thromboembolism events, which require anticoagulation therapy. However, the presence of LAAT requires emergent anticoagulation therapy until the thrombus resolves. The comparable efficacy of DOAC and VKA indicates an opportunity to provide patients with safer prescriptions. This study has several limitations. First, no subgroup analyses were performed for the different classes of DOAC in the 18 studies due to the small number of studies for each DOAC. Further studies are warranted to investigate the effectiveness of different DOACs in patients with different comorbidities. Second, several studies reported adverse effects, bleeding risk, and thromboembolism events, which prevented these events from being included in the meta-analysis. Third, the relative weight of the included studies varied in analyses, which is why the random-effects model was employed. Fourth, although the anti-IIa/Xa activity effectiveness and possible drug–drug interactions are crucial in the issues investigated in the current study, it is not feasible to conduct relevant analyses considering the very limited information provided in the included studies. Another related concern is the ratio/period of the effective therapeutic range, such as the international normalized ratio (INR) values in patients taking VKA. The distinctive ways of reporting INRs in the included studies also hinder further analyses from examining the underlying origins of heterogeneity.

## 6. Conclusions

This meta-analysis of observation data revealed significant differences in LAAT development in high-risk patients and the likelihood of LAAT resolution in patients treated with DOAC or VKA. With respect to safety profiles, DOACs are preferable to VKAs in patients with LAAT and without absolute DOAC contraindication. As the role of DOACs expands, further studies should be conducted to provide clinicians with a practical reference for optimization of the selection of appropriate DOACs and the duration of treatment.

## Figures and Tables

**Table 1:** Baseline characteristics of included studies for systematic review and meta-analysis of the incidence of left arterial appendage thrombus (LAAT) in patients with atrial fibrillation under vitamin K antagonist (VKA) or direct oral anticoagulant (DOAC) treatment.

Author (Year)/ Country, Study Type	Age (Years Old, Mean ± SD), Male (n/%)	CHA2DS2-VASC Score (Mean ± SD)	Anticoagulant (Duration)
Alqarawi (2019)/ Canada, prospective [21]	64 ± 11, (478/72%)	1.9 ± 1.4	VKA (INR ≥ 2) and DOAC: 258 Dab, 184 Riv, and 54 Api (>4 weeks)
Bursi (2021)/ Italy, prospective [22]	71 ± 10, (177/64%)	3.1 ± 1.4	VKA (8.1% INR < 2) and DOAC (>3 weeks)
Durmaz (2020)/Turkey, prospective [23]	69.9 ± 12.4 (LAAT) and 65.1 ± 12.1 (nLAAT), (45.6%)	3.44	VKA (INR not specified) and DOAC (>3 weeks) (61 VKA, 32 Dab, 62 Riv, 29 Api)
Frenkel (2016)/ US, retrospective [24]	65, (287/74%)	2	VKA (INR median 3.0 (IQR: 2.5 to 3.2)) and DOAC: 93 Dab, 62 Riv, and 28 Api (>4 weeks)
Kawabata (2017)/ Japan, retrospective [25]	62 ± 11, (445/79.6%)	1.9 ± 1.5	VKA (INR available in 90% of participants but not specified) and DOAC: 145 Dab, 121 Riv, 40 Api, and 5 Edo (>4 weeks)
Merino (2019)/ Europe, prospective [26]	67.3 ± 9.4 (LAAT) and 64.2 ± 10.8 (nLAAT), (733)	3.0 ± 1.4 (LAAT) 2.7 ± 1.5 (nLAAT)	VKA (INR 1.51 ± 0.61 at baseline) and DOAC (>30 days)
Uziębło-Życzkowska (2020)/Poland, retrospective [27]	63.35, (61%)	2.48 ± 1.53	VKA (INR 1.69 ± 0.86 at baseline) and DOAC (>3 weeks) (VKA 227, 240 Dab, 279 Riv, 4 Api)
Wyrembak (2017)/ US, retrospective [28]	65, (618/66%)	3.1 ± 2	VKA (INR 2.32 ± 0.59) and DOAC (>4 weeks)
Kapłon-Cie’slicka (2022)/ Poland, prospective [29]	67, (1731/63%)	3	VKA (INR not specified) and DOAC (>3 weeks) 814 Dab, 1060 Riv, 388 Api
Karwowski (2022)/ Poland, retrospective [30]	73.4 ± 10.3 (80, 50%)	3.83 ± 1.64	VKA (6 warfarin, 22 acenocoumarol; INR not reported) and DOAC (25 Dab, 83 Riv, 16 Api, and 8 Edo) (>4 weeks)
Feickert (2020)/ Germany, retrospective [31]	71.3 ± 9.0 (68, 48.2%)	4.03 ± 1.53	VKA (INR ≥2, n = 74; INR <2, n = 20) and DOAC (>3 weeks) (32 VKA, 14 Dab, 7 Api, 13 Riv, 1 Edo)
Shiraki (2022)/ Japan, retrospective [32]	65.2 ± 10.1 (193, 26.2%)	nil	VKA (INR not specified) and DOAC (>3 weeks) (120 Dab, 213 Riv, 199 Api, and 108 Edo)
Turek (2022)/ Poland, prospective [33]	65.4 (182, 61.5%)	nil	VKA (INR ≥ 2) and DOAC (>3 weeks) (145 Dab, 80 Riv, 10 Api)

nLAAT, patients without LAAT; Dab, dabigatran; Riv, rivaroxaban; Api, apixaban; Edo, edoxaban; INR, ≥ international normalized ratio.

**Table 2:** Baseline characteristics of included studies for systematic review and meta-analysis of LAAT resolution in patients under VKA or DOAC treatment.

Author (Year), Country/Continent, Study Type	Age, Overall (Mean ± SD)	Male, n (%)	CHA2DS2-VASc (Median/Mean)	Anticoagulant (Type, Duration)
Hao (2015), China, Retrospective [34]	57.7 ± 7.4	36 (87.8)	VKA: 1.41 ± 1.01 Dab: 1.16 ± 1.01	VKA (INR not reported) and Dab (4.2 months, median)
Hussain (2019), US, retrospective [35]	63.2	31 (69)	3.4 ± 1.7	DOAC (60 days, median) VKA (INR not reported; 116 days, median)
Kawabata (2017), Japan, retrospective [25]	64	9 (60)	3.7 ± 1.8	VKA (INR not specified) and 1 Dab (>3 weeks)
Ke (2019), China, prospective [36]	VKA: 64.2 ± 10.5 Riv: 63.7 ± 8.6	66 (82.5)	1.46	VKA (INR not reported) and Riv (12 weeks)
Lip (2015), Europe, prospective (X-TRA) and retrospective (CLOT-AF) [37]	X-TRA: 69.6 ± 11 CLOT-AF: 67.7 ± 9.6	X-TRA: 30 (50) CLOT-AF: 103 (66)	X-TRA: 4.0 CLOT-AF: 3.0	VKA (INR not reported) and DOAC: 12 Dab, 1 Riv, and 7 Api (X-TRA: 6 weeks; CLOT-AF: 3–12 weeks)
Mitamura (2015), Japan, retrospective [38]	67.3 ± 12.7	7 (87.5)	1.88	VKA (INR not reported) and Dab (21–308 days)
Nelles (2021), Germany, retrospective [39]	76.1 ± 8.3	45 (57.7)	4.3 ± 1.1	VKA (INR 2.2 ± 0.2) and DOAC: 15 Dab, 12 Api, 11 Riv and 1 Api (116 ± 79 days)
Niku (2019), Japan, retrospective [40]	71.9 ± 11.9	52 (44)	3.4	VKA (59% had INR values ≥ 2.0) and DOAC: 2 Dab, 12 Riv, and 16 Api (96 ± 72 days)
Wu (2018), US, retrospective [41]	67	33 (75)	3	VKA (INR median 2.7 (IQR 2.2, 3.2) at baseline) and DOAC: 12 Dab, 1 Riv, and 7Api (≥4 weeks)
Yang (2019), China, retrospective [42]	63.5 ± 10.9	52 (72.2)	2	VKA (INR not reported) and DOAC: 26 Dab and 29 Riv (101.5 days)
Mazur (2021) Russia, retrospective [43]	59.7± 9.8	41 (60.3)	2.22 ± 1.40	VKA (INR between 2 and 3) and DOAC (>3 weeks): 14 Dab, 14 Riv, 3 Api
Faggiano (2022) Italy, retrospective [44]	71	175 (66)	4	VKA (INR not reported) and DOAC: 18 Riv, Api 24, Dab 24, Edo 5
Karwowski (2022) Poland, retrospective [30]	76.7 ± 8.2	5 (50)	4.58 ± 1.00	VKA (2 warfarin, 5 acenocoumarol; INR not reported) and DOAC 5 Api

Dab, dabigatran; Riv, rivaroxaban; Api, apixaban; Edo, edoxaban; INR, ≥ international normalized ratio.

## Data Availability

The data presented in this study are available on request from the corresponding authors.

## Supplementary Materials

The following supporting information can be downloaded at: https://www.mdpi.com/article/10.3390/jcdd9100351/s1, Table S1: Search history and results.
