# Supplementary material for: Left Atrial Appendage Thrombosis and Oral Anticoagulants: A Meta-Analysis of Risk and Treatment Response
Source: J Cardiovasc Dev Dis. 2022 Oct 13;9(10):351. doi: 10.3390/jcdd9100351 (PMC9604359; doi:10.3390/jcdd9100351)
Supplement: Supplementary file 1 [file jcdd-09-00351-s001.zip › jcdd-1902487-supplementary.pdf]

Table S1 Search history and results

Database	No. of query	Search terms	Results
Pubmed<br>Final Search Date:<br>2022/07/31	1	(((((((((warfarin[Title/Abstract]) OR (novel oral anticoagulant[Title/Abstract])) OR (oral anticoagulant[Title/Abstract])) OR (anticoagulant[Title/Abstract])) OR (direct oral anticoagulant[Title/Abstract])) OR (vitamin K anticoagulant[Title/Abstract])) OR (non-vitamin K oral anticoagulant[Title/Abstract])) OR (dabigatran[Title/Abstract])) OR (rivaroxaban[Title/Abstract])) OR (apixaban[Title/Abstract])) OR (edoxaban[Title/Abstract]) AND ((left atrial appendage thrombus[Title/Abstract]) OR (left atrial thrombus[Title/Abstract])) AND (atrial fibrillation[Title/Abstract])	186
EMBASE<br>Final Search Date:<br>2022/07/31	1	('warfarin'/exp OR 'warfarin' OR 'novel oral anticoagulant'/exp OR 'novel oral anticoagulant' OR 'oral anticoagulant'/exp OR 'oral anticoagulant' OR 'anticoagulant'/exp OR 'anticoagulant' OR 'direct oral anticoagulant'/exp OR 'direct oral anticoagulant' OR 'vitamin k anticoagulant' OR 'non-vitamin k oral anticoagulant' OR 'dabigatran'/exp OR 'dabigatran' OR 'rivaroxaban'/exp OR 'rivaroxaban' OR 'apixaban'/exp OR 'apixaban' OR 'edoxaban'/exp OR 'edoxaban') AND ('left atrial appendage thrombus':ab,ti OR 'left atrial thrombus':ab,ti) AND 'atrial fibrillation':ab,ti	581
	2	#1 AND ('Article'/it OR 'Article in Press'/it OR 'Conference Paper'/it)	312
Cochrane Central Register of Controlled Trials<br>Final Search Date:<br>2022/07/31	1	'left atrial appendage thrombus' OR 'left atrial thrombus' in Title Abstract Keyword AND 'atrial fibrillation' in Title Abstract Keyword AND 'warfarin' OR 'novel oral anticoagulant' OR 'oral anticoagulant' OR 'anticoagulant' OR 'direct oral anticoagulant' OR 'vitamin k anticoagulant' OR 'non-vitamin k oral anticoagulant' OR 'dabigatran' OR 'rivaroxaban' OR 'apixaban' OR 'edoxaban' in Title Abstract Keyword	118
Google Scholar	1	(((((((((warfarin[Title/Abstract]) OR (novel oral	189

Final Search Date: 2022/07/31		anticoagulant[Title/Abstract])) OR (oral anticoagulant[Title/Abstract])) OR (anticoagulant[Title/Abstract])) OR (direct oral anticoagulant[Title/Abstract])) OR (vitamin K anticoagulant[Title/Abstract])) OR (non-vitamin K oral anticoagulant[Title/Abstract])) OR (dabigatran[Title/Abstract])) OR (rivaroxaban[Title/Abstract])) OR (apixaban[Title/Abstract])) OR (edoxaban[Title/Abstract]) AND ((left atrial appendage thrombus[Title/Abstract]) OR (left atrial thrombus[Title/Abstract])) AND (atrial fibrillation[Title/Abstract])	
